# Complete surgical repair for enlarging coronary aneurysm with coronary-pulmonary artery fistula

**DOI:** 10.1093/icvts/ivae095

**Published:** 2024-05-16

**Authors:** Masaaki Kobayashi, Satoshi Kuroyanagi, Onichi Furuya, Makoto Matsuura

**Affiliations:** Department of Cardiovascular Surgery, Kishiwada Tokushukai Hospital, Osaka, Japan; Department of Cardiovascular Surgery, Kishiwada Tokushukai Hospital, Osaka, Japan; Department of Cardiovascular Surgery, Kishiwada Tokushukai Hospital, Osaka, Japan; Department of Cardiovascular Surgery, Kishiwada Tokushukai Hospital, Osaka, Japan

**Keywords:** Coronary artery, Coronary aneurysm, Coronary-pulmonary artery fistula

## Abstract

A 46-year-old male patient was referred to our hospital due to the presence of a coronary aneurysm showing a tendency to enlarge. Subsequent coronary angiography revealed a diagnosis of coronary aneurysm with a concomitant coronary-pulmonary artery fistula. The patient underwent a successful surgical repair, and postoperatively, experienced an uneventful recovery with no residual shunt or aneurysm.

## INTRODUCTION

Coronary-pulmonary artery fistula (CPAF) is an uncommon coronary artery anomaly characterized by an abnormal connection between the coronary artery and pulmonary artery. Its association with a coronary artery aneurysm is exceedingly rare. Prior research has highlighted the potential for even small coronary artery aneurysms, ∼1 cm in diameter, to rupture, underscoring the significance of considering surgical intervention upon aneurysm confirmation.

## CASE REPORT

**Figure 1:**
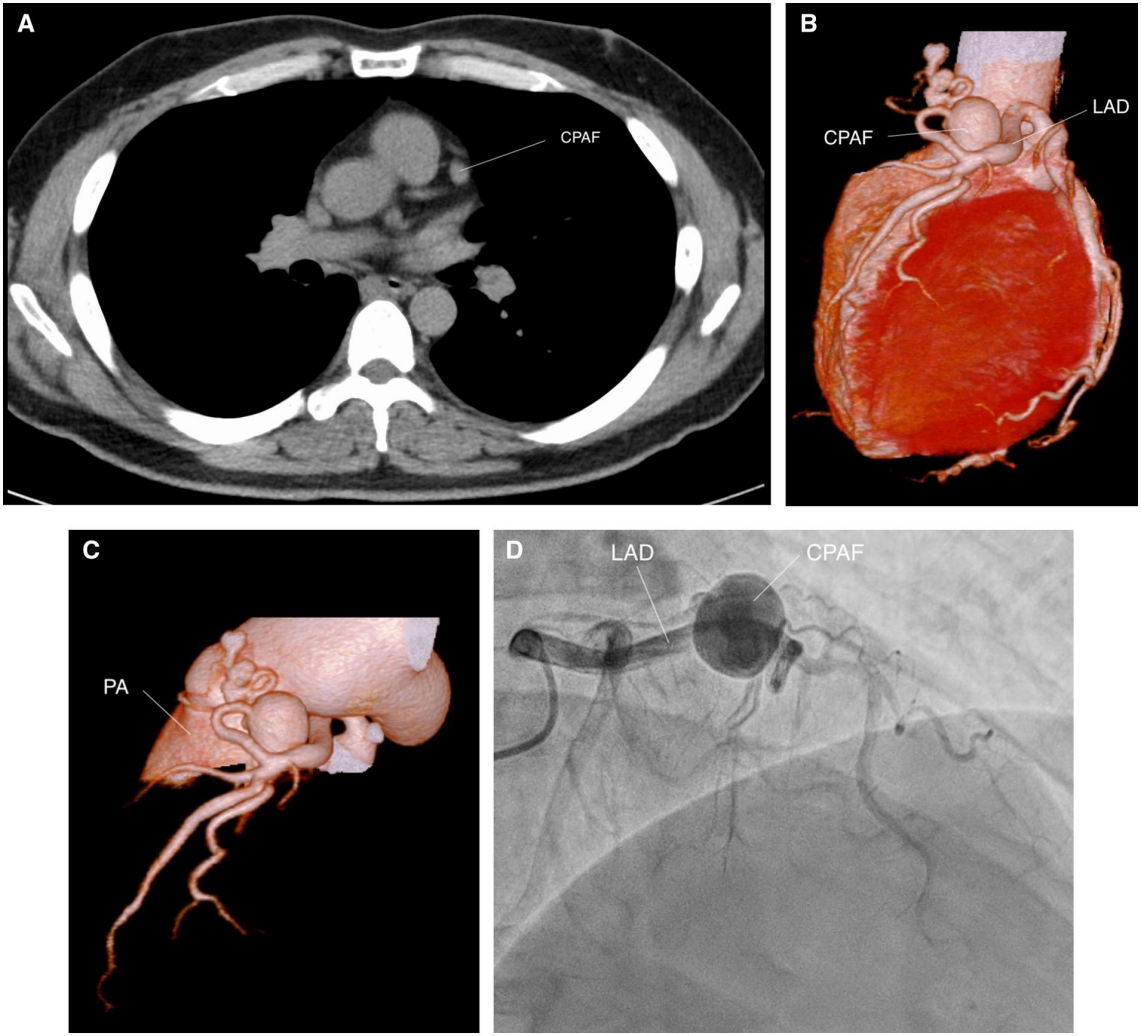
Preoperative images. (**A**) 3 years before, (**B**) 3D CTA, (**C**) CPAF and PA trunk and (**D**) CAG. CAG: coronary angiography; CPAF: Coronary-pulmonary artery fistula; CTA: computed tomography angiography; PA: pulmonary artery.

**Figure 2:**
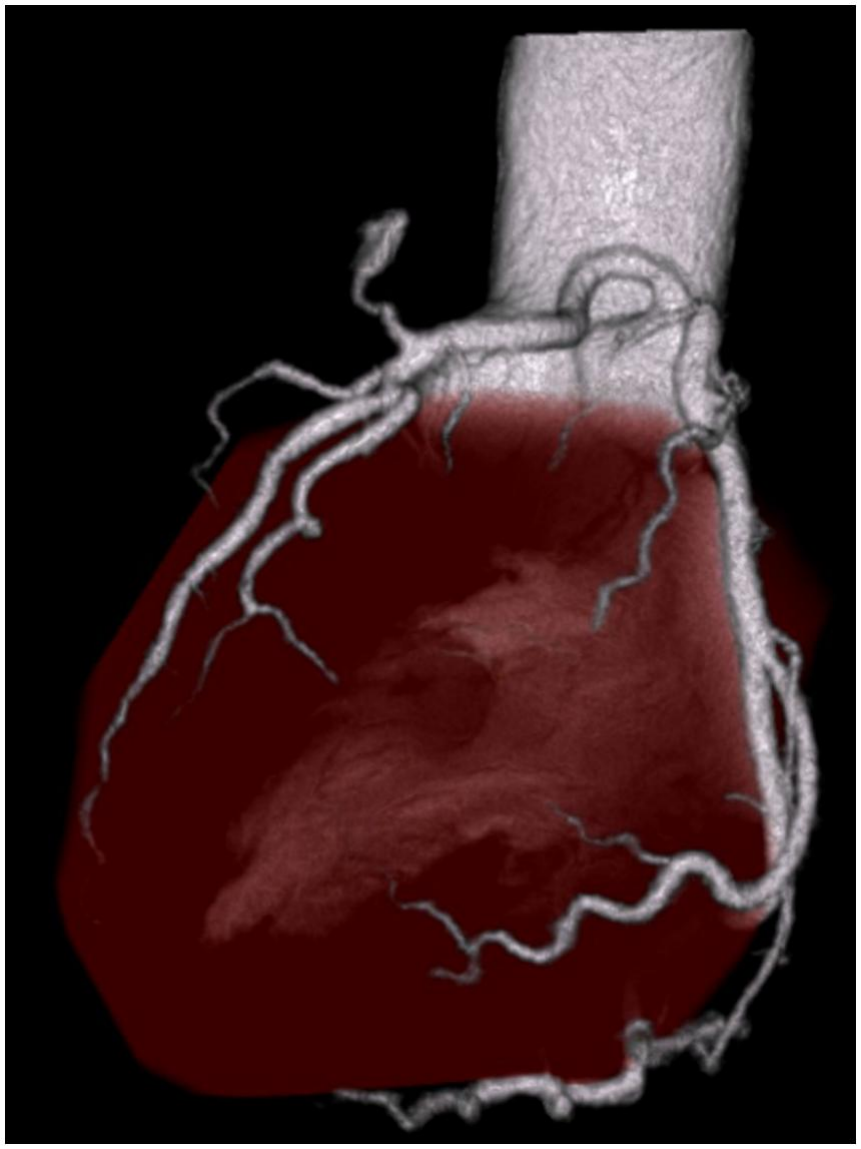
Postoperative CT. CT; computed tomography.

The patient, a 48-year-old male, was referred to us with a coronary artery aneurysm showing an enlarging tendency (Fig. 1A and B) and presented with the following parameters: height (163 cm), weight (73 kg), blood pressure (145/80 mmHg) and pulse (88/min). He was asymptomatic. A contrast computed tomography (CT) scan revealed the presence of a 1.5-cm diameter coronary aneurysm near the pulmonary artery (Fig. 1B and C). Subsequent coronary angiography confirmed the presence of a coronary artery aneurysm with a coexisting CPAF (Fig. 1D). The CPAF was observed to connect from the left anterior descending artery to the left anterior side of the pulmonary artery, appearing closely adhered to the pulmonary artery. Surgical intervention was selected due to considerations regarding the potential risk of aneurysm rupture and to ensure complete treatment without any residual fistula. The surgery was conducted via a median sternotomy approach. Following the establishment of cardiopulmonary bypass with cannulation of the ascending aorta, superior and inferior vena cava, cardiac arrest was induced using an antegrade cardioplegic solution. The aneurysm, which exhibited a thin and soft wall, was located adjacent to the pulmonary artery trunk. The pulmonary artery and the aneurysm were incised during cardioplegia. Two small openings were observed within the aneurysm, and the afferent hole was verified through cardioplegia infusion. Both openings were closed using 4–0 polypropylene sutures with pledgets from the inside. Afterward, we examined the interior of the pulmonary artery and identified a drainage orifice originating from the CPAF within the proximal pulmonary artery trunk. This orifice was also closed using 5–0 polypropylene sutures. Additionally, vessels surrounding the CPAF were ligated from the outside using 5–0 polypropylene sutures. The coronary aneurysm was left open, and a hotshot was performed to ensure the absence of residual blood flow within the aneurysm. After confirming the lack of remaining flow, the aortic cross-clamp was declamped (Video 1). The patient was successfully weaned from cardiopulmonary bypass, with aortic cross-clamp and cardiopulmonary bypass times of 22 and 39 min, respectively. The postoperative course was uncomplicated, and a postoperative CT scan revealed the absence of any residual fistula or aneurysm (Fig. 2).

## DISCUSSION

CPAF is a rare congenital anomaly with an adult prevalence of 1 in 50 000 [1]. Between 19% to 26% of CPAF cases are reported to be associated with an aneurysmal dilatation [2]. Typically, a coronary artery aneurysm larger than 3 cm is an indication for surgery due to the risk of rupture. However, some reports indicate that in patients with a CPAF-related aneurysm, ruptures have been observed in aneurysms sized 1–2 cm [3–5]. The risk of aneurysm rupture in CPAF may be higher than that in native coronary artery aneurysms, likely due to the structural fragility of the fistula. In our case, the coronary artery aneurysm has enlarged from 8 to 15 mm over the course of 3 years. Considering the risk of rupture, we opted for a surgical intervention. Surgery was chosen to ensure a definitive treatment approach. To eradicate the fistula and avoid any residual connections, we not only sutured the aneurysm from the inside but also conducted an intraluminal incision of the pulmonary artery to confirm and ensure the complete closure of the fistula. In cases of CPAF, fistulas may exist that are not discernible from an external view alone. Consequently, performing an incision of the pulmonary artery during cardiac arrest is believed to lead to a definitive closure, ensuring certainty in achieving complete closure.

## CONCLUSION

We report successful surgery for a coronary aneurysm with CPAF.

**Conflict of interest:** none declared.

## Data Availability

All relevant data are within the manuscript and its Supporting Information files.
